# Pandemic preparedness and response in the EMR: adapting lessons learnt from pandemics for tomorrow

**DOI:** 10.3389/fpubh.2025.1653111

**Published:** 2025-08-13

**Authors:** Deema Al Bakri, Andreas Jansen, Edmund Newman, Jonathan Suk, Rana Jawad Asghar, Mouad Merabet, Sarah Hess, Soha Albayat, Yousef Khader, Haitham Bashier, Magid Al-Gunaid, Mohannad Al Nsour

**Affiliations:** ^1^The Eastern Mediterranean Public Health Network, Amman, Jordan; ^2^Federal Information Centre for International Health Protection, WHO Collaborating Centre for GOARN Robert Koch Institute, Berlin, Germany; ^3^Public Health Rapid Support Team, The UK Health Security Agency, London, United Kingdom; ^4^Public Health Emergency Preparedness, European Centre for Disease Prevention and Control, Stockholm, Sweden; ^5^Global Health Strategists and Implementers, Islamabad, Pakistan; ^6^National Center for Emergency Operations, Ministry of Health, Rabat, Morocco; ^7^Epidemic and Pandemic Threat Management Department, World Health Organization HQ, Geneva, Switzerland; ^8^Health Emergency Preparedness, Ministry of Public Health, Doha, Qatar; ^9^Department of Public Health, Jordan University of Science and Technology, Ar-Ramtha, Irbid, Jordan

**Keywords:** emergency preparedness, Eastern Mediterranean Region (EMR), COVID-19, multi-sectoral collaboration, health systems resilience

## Abstract

The Eastern Mediterranean Region (EMR) faces significant public health challenges due to the increasing frequency and impact of pandemics, natural disasters, and ongoing political instability. Despite progress in areas such as vaccination rates, surveillance, and laboratory capacities, gaps in pandemic preparedness persist, underscoring the need for comprehensive, multi-sectoral strategies. The Eastern Mediterranean Public Health Network (EMPHNET) organized a roundtable during its Eighth Regional Conference to examine key aspects of pandemic preparedness and response, focusing on lessons learned from recent health crises like COVID-19. The session highlighted critical themes, including the importance of sustainable investments in preparedness, addressing health workforce shortages, fostering multi-sectoral collaboration, and prioritizing equity. Panelists and participants emphasized the necessity of trust-building and community engagement as foundational elements for effective health interventions. Discussions also explored innovative frameworks, such as the WHO’s Preparedness and Resilience for Emerging Threats (PRET) initiative, which integrates adaptable and transmission-based approaches. The roundtable concluded with a call for stronger leadership, enhanced governance, and institutionalized learning mechanisms to ensure that lessons from past crises are effectively integrated into future strategies. By transitioning from reactive responses to proactive preparedness, the EMR can build resilient health systems capable of managing future public health emergencies and advancing regional and global health security.

## Introduction

Over the past two decades, there has been an alarming increase in the frequency of primary and secondary health threats and their impact on human life and the economy, with developing countries being affected considerably more than developed countries (1). Besides disease outbreaks like cholera, COVID-19, and measles, the East Mediterranean Region (EMR) has been impacted by natural disasters that increase the threats to public health emergencies of international concerns (2).

In the case of the COVID-19 pandemic, unprecedented challenges were presented, which highlighted the imperative need for efficient pandemic preparedness and response. This need has become more important now than ever. The challenges of the COVID-19 pandemic and other epidemics have insightful lessons that can help create a more resilient future (2).

Public health emergencies in the EMR are further complicated by political instability, protracted conflicts, and the displacement of millions of people, which places immense pressure on already fragile healthcare systems (3). The EMR is distinguished by distinctive socio-cultural dynamics and varied healthcare systems (4). These systemic vulnerabilities need a unified, multi-sectoral approach to pandemic preparedness and response that transcends beyond borders and prioritizes health equity (5). The region’s diverse socio-economic and cultural contexts also provide unique opportunities for innovation and tailored strategies that can address both immediate and long-term health threats.

Following COVID-19, according to World Health Organization (WHO), countries in the EMR have become more prepared for managing public health emergencies (6). This is evident by the improvements in vaccination rates, surveillance and data management, and laboratory capacities (6). However, there is still a need for improvement, for example, strengthening health systems and tackling inequalities in access to healthcare (7, 8).

The Eastern Mediterranean Public Health Network (EMPHNET) remains committed to supporting countries’ preparedness to emergencies through many of its programs and mainly through its Public Health Emergency Management Center (PHEMC). As we stand at the intersection of recovery and readiness, EMPHNET has organized a roundtable titled “Pandemic Preparedness and Response: Adapting Lessons for Tomorrow” to explore these aspects, fostering collaboration to strengthen resilience and mitigate future risks by bringing together experts, decision-makers, and other relevant stakeholders and audiences.

The roundtable aimed to explore key challenges, opportunities, and lessons in pandemic preparedness and response in the EMR, drawing from recent experiences including the COVID-19 pandemic. It also sought to foster dialogue among global and regional experts on practical approaches to strengthening future readiness. This paper aims to document the key insights and recommendations shared during the session and reflect on their implications for advancing resilient and equitable preparedness frameworks in the region.

### Methods

The roundtable session lasted 2 h and featured six expert presentations. Notes were taken by members of the EMPHNET technical team throughout the session to capture key points, speaker insights, and audience questions. Following the event, the content was reviewed and synthesized by the authors into thematic areas that reflected the major topics discussed, including investment, equity, leadership, workforce development, and community engagement. This paper presents a summary of those discussions and the key recommendations that emerged.

### Roundtable description

The roundtable was conducted in English and included six expert talks highlighting key areas followed by an interactive question and answer (Q&A) session moderated by Dr. Soha Albayat, Director of Health Emergency Preparedness, Ministry of Public Health, Doha, Qatar.

It provided participants with a comprehensive exploration of Public Health emergency preparedness and strategies for their prevention and control in the EMR. Around 80 individuals participated, including International Health Regulations (IHR) focal points, One Health experts, Ministerial officials, public health professionals, Field Epidemiology Training Program (FETPs), Rapid Response Teams (RRTs), health experts interested and non-governmental bodies, and other health professionals.

Dr. Andreas Jansen, Director of the WHO Collaborating Centre for the Global Outbreak Alert and Response Network (GOARN) and Head of the Information Centre for International Health Protection at the Robert Koch Institute (RKI), opened the discussion by discussing the critical role of workforce preparedness in addressing public health emergencies. Drawing on his extensive experience in coordinating outbreak responses across multiple countries, Dr. Jansen highlighted the persistent gaps in health workforce capacities, which remain a significant challenge despite high-level commitments.

Dr. Edmund Newman, Director of the UK Public Health Rapid Support Team (UK-PHRST) and an esteemed expert in infectious disease outbreak response, focused his discussion on the challenges and lessons from public health interventions during complex emergencies in the EMR. He highlighted the increasing complexity of public health responses, emphasizing the interplay of health, humanitarian, and logistical challenges in regions marked by conflict, displacement, and natural disasters. The UK-PHRST has focused on fostering partnerships and building capacity within local health systems to ensure sustainable impact.

Dr. Jonathan Suk, Principal Expert in Emergency Preparedness and Response at the European Centre for Disease Prevention and Control (ECDC), contributed insights from the European experience in translating pandemic lessons into concrete action. He emphasized the importance of operationalizing preparedness through national action plans and revised pandemic preparedness strategies grounded in evidence. Dr. Suk highlighted the critical role of cross-border cooperation, simulation exercises, and technical guidance in strengthening regional resilience, particularly through ECDC’s work under the EU Initiative on Health Security with Southern Neighborhood Policy countries.

Sarah Hess, Unit Head of Pandemic Preparedness Global Platforms at the World Health Organization (WHO), contributed to the roundtable by focusing on the global frameworks essential for pandemic preparedness and response. Ms. Hess introduced WHO’s innovative “Preparedness and Resilience for Emerging Threats” (PRET) initiative, which advocates a transmission-based approach to pandemic preparedness. This framework leverages existing systems to create efficiencies, ensuring that countries are better equipped for the inevitable occurrence of another respiratory or other pandemic.

Dr. Mouad Merabet, Coordinator of the National Public Health Emergency Operations Center within Morocco’s Ministry of Health and Social Protection, presented Morocco’s experience in pandemic and epidemic preparedness, emphasizing the pivotal role of field epidemiology. Reflecting on Morocco’s response to the 2023 earthquake, Dr. Merabet showcased the indispensable contributions of field epidemiologists in rapid response, risk assessments, and situation analysis during emergencies. The deployment of trained personnel to affected areas enabled rapid disease surveillance and timely health interventions, helping prevent secondary health crises amid the disaster. This event underscored the importance of integrating emergency preparedness within broader disaster response strategies.

Dr. Rana Jawad Asghar, a US Epidemic Intelligence Service (EIS) alumnus and the Chief Executive Officer of Global Health Strategists & Implementers, concluded the roundtable with a comprehensive overview and commentary based on the discussions. Drawing from his extensive experience, including establishing Pakistan’s Field Epidemiology and Laboratory Training Program (FELTP), Dr. Asghar critically analyzed the challenges and lessons highlighted by earlier speakers. He agreed that the global health community has yet to learn essential lessons from COVID-19. Using data from his own published research, he underscored the financial disparity between the estimated costs of pandemic preparedness and the actual investments made globally, which he linked to preventable economic losses during COVID-19.

The presentations were followed by a Q&A session, where participants discussed challenges, opportunities, and recommendations for enhancing pandemic preparedness and response in the region.

## Findings

### Inadequate investments in pandemic preparedness

The lack of sufficient investment in pandemic preparedness was a key concern discussed during the roundtable. Dr. Asghar emphasized the alarming disparity between the annual cost of preparedness, estimated at $8–10 billion, and the actual investments made globally. He pointed out that insufficient funding undermines critical areas like infrastructure development, workforce training, and public health systems. Dr. Soha Al-Bayat echoed this sentiment, emphasizing the necessity of shifting focus from reactive to proactive funding models to avoid catastrophic economic losses like those seen during COVID-19. Similarly, Dr. Hess emphasized the lack of financial prioritization for public health preparedness and the need for frameworks to hold countries accountable for their commitments.

Furthermore, Dr. Asghar commented on the evaluation frameworks like the Joint External Evaluations (JEE) can be over-complicated and fail to provide actionable insights on preparedness. He advocated for a streamlined approach that prioritizes critical areas and links funding to measurable outcomes. These comments reinforced the urgent need for global leaders to prioritize pandemic preparedness over other less urgent expenditures.

Dr. Soha Al-Bayat highlighted the consequences of insufficient funding, noting that it directly impacts the ability to train healthcare workers, improve surveillance systems, and strengthen laboratory capacities. She reinforced that governments often prioritize immediate crises over building long-term resilience, a cycle that leaves nations vulnerable to future health threats.

### Health workforce gaps and surge capacity challenges

The health workforce was identified as a critical weakness in pandemic preparedness, particularly in the EMR. Dr. Jansen revealed that despite high-level discussions on the importance of workforce development, tangible progress has been minimal. The region faces persistent shortages of healthcare professionals, and existing personnel often lack the specialized training needed to respond to complex public health emergencies. These gaps are further strained during pandemics when demand for healthcare services surges, often overwhelming systems. He highlighted the importance of leveraging regional networks like GOARN to improve surge capacities and addressing these gaps. However, he argued that relying on external support is not a sustainable solution. Investing in local training programs, particularly for field epidemiologists, is critical for building a self-reliant and resilient workforce capable of responding to future health emergencies. Dr. Asghar added that many health systems lack the foundational positions for epidemiologists, which hampers their ability to respond effectively during crises.

### Need for multi-sectoral collaboration

Pandemic preparedness cannot succeed in isolation; it requires robust collaboration across health, social, and economic sectors. Dr. Suk highlighted how zoonotic diseases often emerge from agricultural practices, underscoring the need for closer ties between public health and agricultural sectors. He urged that addressing root causes of pandemics, such as land use and animal-human interactions, requires coordinated efforts that go beyond the health sector.

Sarah Hess added that collaboration must be embedded in policy frameworks, ensuring that sectors like labor, faith, education, and agriculture are actively involved in preparedness planning. These discussions highlighted the importance of leadership in driving collaborative efforts and creating unified strategies. Both speakers agreed that fostering collaboration requires strong governance structures and clear accountability mechanisms. They advocated for embedding cross-sectoral partnerships in preparedness plans to address the root causes of pandemics and create resilient systems capable of managing complex crises. Dr. Soha supported this perspective, noting that successful pandemic preparedness relies on strong leadership to foster multi-sectoral collaboration. She pointed out that breaking down silos between sectors is not only necessary but also challenging, as it requires consistent engagement and trust-building during non-crisis periods.

### Importance of community engagement and trust building

Community engagement was identified as a cornerstone of successful pandemic response. Dr. Suk highlighted how many public health measures fail because they are not designed with community input or cultural relevance. For example, during COVID-19, mistrust in governments and institutions often led to poor compliance with public health measures. Dr. Sarah Hess reinforced this point, stressing that trust cannot be built during a crisis, but rather requires sustained efforts over time. She highlighted the importance of two-way communication that listens to community concerns and incorporates their feedback into policies. Long-term investments in community engagement not only build trust but also strengthen resilience, ensuring communities are active partners in emergency responses.

Social scientists and anthropologists were recognized as vital contributors to this process, as their expertise helps design culturally appropriate interventions that resonate with communities. The panel agreed that building trust requires long-term investment but yields significant dividends in resilience and compliance during emergencies.

### Challenges in leadership and governance

Leadership was a recurring theme, with participants emphasizing its central role in pandemic preparedness. Ms. Hess highlighted how gaps in decisive leadership during COVID-19 undermined response efforts, particularly in managing uncertainty and coordinating multi-sectoral activities. Dr. Asghar added that governments often hesitate to seek international support, fearing it could be perceived as a failure, which delays critical interventions and exacerbates crises. He advocated for leadership training programs that include crisis management and advocacy skills, enabling leaders to navigate complex emergencies effectively. Both speakers underscored that strong leadership is essential to drive collaboration, secure resources, and implement preparedness plans effectively.

Dr. Soha emphasized that effective leadership requires more than technical expertise; it demands the ability to communicate transparently, build coalitions, and advocate for resources. The panel concluded that strengthening leadership at all levels is essential for improving pandemic preparedness and response capabilities.

### Equity in pandemic preparedness and response

Equity was a major focus of the discussions, with participants highlighting how pandemics disproportionately affect vulnerable populations. Dr. Suk and Dr. Newman pointed out that marginalized communities often have limited access to healthcare and resources, leaving them more exposed to public health emergencies. For instance, vaccine distribution during COVID-19 was frequently inequitable, with high-income countries securing the majority of doses while low-income regions struggled to vaccinate their populations.

The panel emphasized that addressing inequities requires both targeted interventions and systemic changes and have called for prioritizing underserved areas in vaccination campaigns and healthcare delivery, as well as empowering local responders to take the lead in community-level initiatives. Participants agreed that international agencies and NGOs play a crucial role in promoting equity by supporting resource-constrained governments and advocating for fair distribution of resources.

### Adapting lessons learned into practical frameworks

While the COVID-19 pandemic provided valuable lessons, participants noted that translating these lessons into actionable frameworks remains a challenge. Dr. Asghar criticized the expansion of JEE indicators, arguing that the added complexity does not improve preparedness outcomes. Instead, he advocated focusing on a few critical priorities and ensuring accountability in their implementation.

Ms. Hess introduced the WHO’s Preparedness and Resilience for Emerging Threats (PRET) initiative, which adopts a transmission-based approach to pandemic preparedness and planning. This framework leverages existing systems to address multiple pathogens, creating efficiencies and reducing duplication. Dr. Soha praised PRET as a promising step forward but cautioned that its success depends on strong leadership and adequate funding. The panel agreed that practical, adaptable frameworks like PRET are essential for translating lessons learned into effective preparedness strategies.

The 2023 earthquake in Morocco served as a powerful reminder of the need for integrated emergency preparedness frameworks that go beyond health-specific scenarios. During the roundtable, Morocco’s response was highlighted as an example of how pre-established field epidemiology capacity enabled rapid public health assessments and timely interventions amidst a complex humanitarian emergency. The deployment of trained personnel facilitated effective surveillance and coordination between national and local actors, helping to prevent the escalation of secondary health crises. This experience underscored the importance of embedding adaptable public health preparedness systems within broader disaster risk management strategies, particularly in regions facing intersecting natural and health-related threats.

## Discussion

The findings from the roundtable underscored a critical gap in the global response to pandemics: while many lessons have been learned from past experiences, including COVID-19, others remain unacknowledged or forgotten. For example, the emergence of Mpox, caused by the Monkeypox virus (MPXV) highlighted a failure to apply critical insights from COVID-19, particularly in addressing stigma and ensuring equitable access to healthcare. Despite recognizing the harmful effects of stigma during the HIV/AIDS epidemic and its resurgence during COVID-19 against specific ethnic groups, similar discriminatory narratives emerged during the Mpox outbreak (9). These stigmas not only marginalized affected populations but also delayed reporting and hindered effective public health responses (10).

Another forgotten lesson is associated with disproportionate reliance on reactive measures, such as border closures, rather than proactive preparedness strategies. During the COVID-19 pandemic, it became evident that border closures, while sometimes necessary, are often ineffective if implemented too late and without complementary measures like robust surveillance and contact tracing (11). This cycle of “learn and forget” underscores the need for institutionalized memory within public health systems in a mechanism to document, disseminate, and operationalize lessons learned to prevent the repetition of past mistakes.

These examples highlight the urgent need for sustained investment in education, advocacy, and structural reforms to ensure that lessons from past crises are integrated into future responses. This includes fostering cultural sensitivity in health communication, developing clear protocols to avoid over-reliance on reactive measures, and prioritizing equity in all aspects. Multi-sectoral collaboration is essential for comprehensive pandemic preparedness (12). Zoonotic diseases, which account for the majority of emerging infectious diseases, often result from human-animal-environment interactions (13), underscoring the need for an integrated One Health approach. Studies highlight the importance of breaking down silos across sectors such as agriculture, environment, and public health to address the root causes of pandemics (14). Effective governance frameworks and clear accountability mechanisms are key to fostering collaboration and ensuring that all sectors are actively engaged in preparedness planning of pandemic preparedness and response (15).

The global underinvestment in pandemic preparedness is a recurring challenge that directly impacts the ability to prevent and mitigate public health crises. Despite clear evidence that proactive investments yield significant economic and health benefits, funding gaps persist (16). Global spending remains far below this threshold. These financial shortfalls undermine critical components such as infrastructure development, workforce training, and surveillance systems, leaving nations vulnerable to crises like COVID-19. Current evaluation frameworks, such as the Joint External Evaluations (JEE), are often criticized for their complexity and lack of actionable focus (17). There is a growing consensus on the need for streamlined, outcome-oriented frameworks that link funding to measurable improvements in preparedness.

Health workforce gaps represent another critical area requiring attention. The global shortage of healthcare workers, particularly in low- and middle-income countries, exacerbates the strain on health systems during emergencies (18). Research underscores the importance of surge capacity and regional collaboration in addressing these gaps (19). Networks such as GOARN have demonstrated the value of cross-border cooperation in enhancing response capabilities (20). However, sustainable solutions necessitate investing in local training programs, particularly in the field of epidemiology, to build a self-reliant workforce capable of responding effectively to future health emergencies.

Community engagement and trust-building are fundamental to successful public health interventions (21). Public trust in governments and health agencies directly influences compliance with health measures, as evidenced during the COVID-19 pandemic. Poor communication and culturally inappropriate interventions often lead to mistrust and resistance. Long-term community engagement plays a vital role in building social trust and strengthening resilience, particularly in public health crisis responses. Trust-building, as a people-centered approach, fosters active engagement among individuals, communities, and institutions, creating resilient communities capable of effectively preparing for and responding to emergencies (21).

Leadership and governance challenges remain a significant barrier to effective pandemic preparedness. Decisive, transparent, and accountable leadership is essential for managing uncertainties and coordinating multi-sectoral responses. Delays in decision-making and reluctance to seek international support often exacerbate crises. Leadership training programs that integrate advocacy, crisis management, and coalition-building skills can empower leaders to navigate complex emergencies effectively and implement robust preparedness plans (22).

Equity in pandemic preparedness and response continues to be a critical issue. Vulnerable populations, including marginalized communities and low-income regions, are disproportionately affected during pandemics due to inequities in resource distribution and healthcare access (23). Addressing these disparities requires targeted interventions, such as prioritizing underserved areas in vaccination campaigns and investing in community-led initiatives (24). Research supports the role of international partnerships in promoting equity by providing financial and technical support to resource-constrained countries.

The roundtable findings highlighted a major weakness in the global pandemic response efforts: a cycle of “learning and forgetting” underscores the need for institutionalized memory within public health systems. This calls for mechanisms to document, disseminate, and apply lessons learned to avoid the repetition of past mistakes.

## Conclusion

Pandemic preparedness and response in the EMR must evolve to meet the unique challenges posed by the region’s socio-cultural dynamics, political instability, and health system vulnerabilities. The lessons learnt, not learnt, or even forgotten from recent pandemics, particularly COVID-19, emphasize the need for comprehensive strategies that integrate robust public health systems, multi-sectoral collaboration, and equity-focused interventions. Strengthening surveillance, laboratory capacities, workforce training, and community trust-building are essential pillars of preparedness.

The roundtable on *Pandemic Preparedness and Response in the EMR: Adapting Lessons Learnt from Pandemics for Tomorrow* highlighted these priorities while providing a platform for experts to share insights and explore innovative solutions. The discussions emphasized the importance of adopting adaptable frameworks that leverage existing systems and focused on actionable priorities. It also underscored the critical role of leadership, sustainable investment, and institutionalized learning to ensure that lessons from past crises inform future strategies effectively.

The path forward requires a shift from reactive responses to proactive preparedness, supported by stronger governance structures, enhanced collaboration across sectors, and dedicated efforts to address inequities. The EMR can develop resilient health systems capable of withstanding future public health emergencies, protecting the most vulnerable, and fostering regional and global health security by embracing these approaches.

## Data Availability

The original contributions presented in the study are included in the article/supplementary material, further inquiries can be directed to the corresponding author.
